# Patient values in physiotherapy practice, a qualitative study

**DOI:** 10.1002/pri.1877

**Published:** 2020-09-11

**Authors:** Carla M. Bastemeijer, Johannes P. van Ewijk, Jan A. Hazelzet, Lennard P. Voogt

**Affiliations:** ^1^ Department of Public Health Erasmus University Medical Center Rotterdam The Netherlands; ^2^ University of Humanistic Studies Utrecht The Netherlands; ^3^ Department of Physical Therapy Studies Rotterdam University of Applied Sciences Rotterdam The Netherlands

**Keywords:** patient‐centred care, patient preference, primary healthcare, professional–patient relations, qualitative research

## Abstract

**Objectives:**

Physiotherapy is, like all healthcare professions, relational and value‐laden. Patient‐centred care, evidence‐based practice and value‐based practices are concepts in which patient values lie at the heart of high‐quality healthcare practices. Nevertheless, physiotherapists have limited awareness of what patient values are in the physiotherapy encounter. The purpose of this study is to explore these patient values.

**Methods:**

A qualitative study design using content analysis was used involving 17 adult participants with chronic or recurrent musculoskeletal pain. Data were collected during July 2015–July 2016 in three primary care physiotherapy facilities in Rotterdam, The Netherlands. Two researchers analysed the interviews and derived relevant codes from the data. After an iterative process of comparing, analysing, conceptualizing, and discussing the data, a pre‐existing analytic framework was refined in which distinct values were delineated.

**Results:**

Emerging patient values were encompassed in three themes, each consisting of two to four elements: (1) values about oneself (uniqueness and autonomy), (2) values regarding actions of the professional (technically skilled professional, conscientious professional, compassionate professional, responsive professional) and (3) values regarding interactions between patients and the professionals (partnership and empowerment).

**Conclusion:**

This study emphasizes the need for discussing patient values in the clinical encounter and helps physiotherapists to understand what deems to be important for patients with musculoskeletal pain in physiotherapy practice. The results of this study contribute to the existing body of knowledge of this important aspect of the quality of physiotherapy practice and may inspire clinicians and educators to actively implement patient values in clinical practice and the physiotherapy education.

## 1 INTRODUCTION

Physiotherapy is, like all healthcare professions, deeply relational and value‐laden. It can be characterized by the nature of complex interactions between physiotherapists and their patients. This complexity results from the often multifactorial nature of health problems, the limited evidence base of physiotherapeutic interventions and the unique and personal contextual aspects of the personal health problem. The clinical encounter is the place where the separate worlds of patient and healthcare professional meet and ideally merge. This merging of professional and layman knowledge, professional and patient experiences and professional and patient values is, however, not straightforward. Physiotherapists experience tensions between the choice of treatment they feel is best for their patients and the beliefs and attitudes of patients themselves (Jeffrey & Foster, 2012). In their turn, patients in physiotherapy practice often experience a lack of feeling believed or being understood by their physiotherapists (Harding, Parsons, Rahman, & Underwood, 2005; Potter, Gordon, & Hamer, 2003; Toye & Barker, 2012; Trede, 2012). This lack of understanding can be the consequence of limited attention of healthcare professionals for the personal needs and values of their patients (Kennedy et al., 2017). One reason for this may the fact that the meaning of the concept of patient values is currently incomplete, too abstract and/or undertheorized (Charles, Gafni, & Freeman, 2011; Kelly, Heath, Howick, & Greenhalgh, 2015; Rosenbaum, 2013).

Values are basic principles that individuals, groups or societies have about what is deemed to be good, bad or desirable (Moyo, Goodyear‐Smith, Weller, Robb, & Shulruf, 2016). They are formed from an early stage in life and are further shaped by life events, social contacts and education (Schwartz, 2012). Rokeach defines values as enduring beliefs that influence a specific mode of conduct or end state of existence that provide us with our moral framework (Rokeach, 1973). Patient values are parts of the concepts ‘evidence‐based practice’ (EBP), ‘value‐based practice’ (VBP) ‘patient‐centred care’ (PCC) and are also embodied in the declaration of Helsinki (Fulford, Peile, & Carroll, 2012; IOM, 2001; Sackett, Rosenberg, Gray, Haynes, & Richardson, 1996; WMA, 2001). In these concepts, patient values are considered to lie at the heart of high‐quality healthcare practices and underscore the importance to consider aspects that people value in healthcare practices such as being taken seriously, being treated by a competent professional, feeling safe and being involved in decision‐making (Bernhardsson, Larsson, Johansson, & Öberg, 2017; Entwistle, Firnigl, Ryan, Francis, & Kinghorn, 2012; Schoot, Proot, Meulen, & Witte, 2005; Skea, MacLennan, Entwistle, & N'dow, 2014). This latter meaning of patient values refers to people's preferences and expectations regarding medical interventions or procedures.

Results of our earlier systematic review of qualitative studies regarding the content and meaning of the concept of patient values, shows that patient values in healthcare can be divided into three categories: (1) values concerning the life and philosophy of the patient, such as the wish for autonomy and the desire to be considered a unique person; (2) values related to the characteristics and behaviour of the professional, such as being responsive, compassionate and professional and (3) values regarding the relationship between the patient and the professional, such as the wish for partnership and empowerment (Bastemeijer, Voogt, van Ewijk, & Hazelzet, 2017). Until now, it remains unknown how these values and expectations merge (or not) with professional interpretations of complex health problems and shape clinical encounters in physiotherapy.

The aim of this study is to describe the aspects of physiotherapy practice that people with musculoskeletal pain value in high‐quality care. These findings will be used to further develop our earlier found taxonomy of patients values in healthcare.

## 2 METHODS

### 2.1 Design

This study is designed as an explorative qualitative study using content analysis (Krippendorff, 2018). The 32‐item consolidated criteria for reporting qualitative research checklist is used to design and report the study (Tong, Sainsbury, & Craig, 2007).

### 2.2 Participants and setting

Seventeen participants were recruited from three primary care physiotherapy practices in Rotterdam, The Netherlands. These sites were assumed to be high‐quality care practices judged by independent auditors. The participants were asked to enrol into this study by their physiotherapist, who was instructed regarding inclusion criteria by the principal investigator (CB). Participants were eligible if they sought consultation for chronic or recurrent musculoskeletal low back, neck and shoulder pain. Those are among the most prevalent pain problems in primary care, known for their complex biopsychosocial character and therefore appropriate health problems suited for the aim of this study (Jordan et al., 2010). Purposive sampling was used to achieve variation in terms of gender, age and level of education given the fact that values have been formed during life, by personal life events, social contacts and education (Schwartz, 2012). From each location, five to seven participants were recruited of which nine were female and eight were male, aged between 33 and 79 years (57 years on average). Eleven of the participants suffered from chronic or recurrent low back or neck pain and six of them from shoulder pain. Nine participants had at least tertiary education (Table 1; Characteristics of participants). Participants were informed about the aim of the study and received written information about participating in medical scientific research prior to the start of the study. After 5 days, eligible participants were contacted by the principal researcher (CB) by telephone for definitive enrolment into the study. An interview was scheduled at a location of their choice.

**TABLE 1:** Characteristics of participants

Participant	Sex	Age	Education	Musculoskeletal condition	Experience with physiotherapy
P01	F	62	≥Tertiary education	Shoulder pain	Over 20 years intermittent
P02	F	71	<Tertiary education	Shoulder pain	Over 20 years intermittent
P03	F	66	≥Tertiary education	Shoulder pain	One and a half year
P04	M	55	≥Tertiary education	Low back pain	Over 20 years intermittent
P05	F	33	<Tertiary education	Neck pain	3 years intermittent
P06	F	61	≥Tertiary education	Neck pain	Over 20 years intermittent
P07	M	69	≥Tertiary education	Shoulder pain	Over 10 years intermittent
P08	F	44	≥Tertiary education	Low back pain	1 year
P09	M	65	<Tertiary education	Shoulder pain	Over 10 years intermittent
P10	F	48	<Tertiary education	Low back pain	Over 7 years intermittent
P11	F	42	≥Tertiary education	Neck pain	Over 10 years intermittent
P12	F	44	≥Tertiary education	Shoulder pain	Over 30 years intermittent
P13	M	70	<Tertiary education	Neck pain	Over 30 years intermittent
P14	M	79	<Tertiary education	Low back pain	Over 30 years intermittent
P15	M	46	<Tertiary education	Low back pain	Over 10 years intermittent
P16	M	71	<Tertiary education	Low back pain	Over 10 years intermittent
P17	M	48	≥Tertiary education	Low back pain	Over 10 years intermittent

Abbreviations: F, female; M, male.

### 2.3 Data collection

Following two pilot interviews, which were discussed by all authors regarding scope and sufficient depth, 17 face‐to‐face open interviews were conducted between July 2015 and July 2016. The interviews were executed by the principal researcher (CB), a practicing physiotherapist and PhD‐researcher with 20 years clinical expertise in primary care as a physiotherapist and with post‐graduate training in qualitative research methods. There was no prior relationship with the participants.

Based on the results of the systematic review on the content of patient values in healthcare practices, three topics were addressed in the interviews: (1) personal values of patients regarding humanity and physiotherapy care; (2) patients' values regarding actions and behaviour of the physiotherapist and (3) the patients' values regarding the interaction with the physiotherapist (Bastemeijer et al., 2017). These topics and other (non‐)related topics were discussed in depth and participants were encouraged to illustrate their thoughts with lived experiences. Data collection was ended when saturation was obtained (where the last three interviews contribute little or no new understandings) (Gentles, Charles, Ploeg, & McKibbon, 2015). The interviews lasted 35–64 min (53 min on average), were audiotaped and transcribed verbatim by an independent administrative assistant. All participants verified the verbatim of their own interview as part of a member checking process. The findings and quotes were completely anonymized by the interviewer.

### 2.4 Data analysis

Content analysis as described by the procedures and criteria of Krippendorf was used to explore the acquired data (Krippendorff, 2018). The unit of analysis was the transcriptions of interviews. To familiarize the researchers to the transcripts and audio files, two researchers (CB and LV) separately read and re‐read the interviews to code meaningful words, sentences or paragraphs. Both manifest (analytical) content and latent (interpretative) content was analysed. Subsequently, both researchers discussed their mutual interpretations and together formed a shared understanding of the data. Then they investigated whether analytical and interpretative (sub‐)elements that arose by discussing and organizing the initial codes could be organized in the themes and elements as found in our earlier review (Bastemeijer et al., 2017). Data points that were ambiguous or non‐placeable were discussed by the two primary investigators to determine appropriate organization within the (sub‐)elements which helped to sort the experiences of patients with physiotherapy practice. All aforementioned steps of the analysis were discussed with the whole research team in order to prevent (unconscious) bias, to verify the analysis and provide analyst triangulation. Atlas.ti was used for data management and further organization and interpretation of the themes, elements and sub‐elements (Friese, 2013).

Trustworthiness of the study was addressed by enhancing credibility, dependability, conformability and transferability (Trochim & Donnelly, 2007). Credibility was addressed by the method of data collection (open interviews), thus allowing participants to express a variety of perceptions, experiences and values. The member checking process with regard to the transcripts and summarizing the findings during and after the interview allowed participants to verify interpretations. Furthermore, the data were peer‐checked by two authors (CB and LV) during the analytical process to reduce risk of bias. Data were triangulated by obtaining and comparing data of former research in this field (Bastemeijer et al., 2017; Entwistle et al., 2012; Schoot et al., 2005; Skea et al., 2014; Wijma et al., 2017). Dependability was addressed by the assessment of pilot interviews by all authors. Carefully documenting the steps and choices in the whole research process obtained transparency. The interviews were recorded and the data were anonymously processed during research in Atlas.ti. Confirmability was enhanced by maintaining field notes during the process of interviewing and memo writing during the analysis process. Transferability was achieved by providing clear descriptions of the participants, setting, data collection and data analysis.

## 3 RESULTS

**FIGURE 1:**
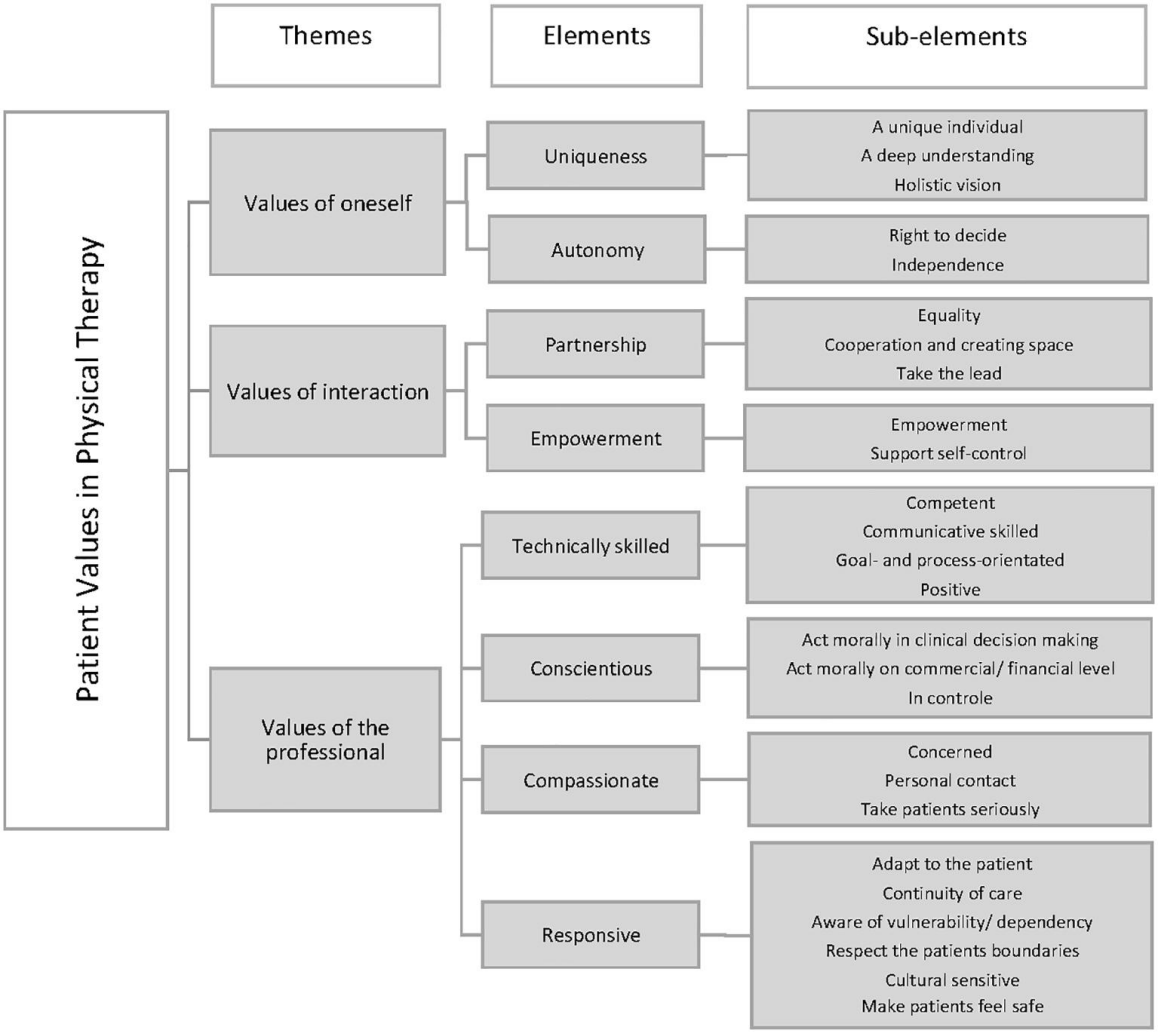
Results

A previously designed scheme (Bastemeijer et al., 2017) was used to organize the data into three themes: (1) values of oneself, (2) values of the professional and (3) values of interaction. Two to four elements per theme were identified (1) uniqueness, (2) autonomy, (3) technically skilled professional, (4) conscientious professional, (5) compassionate professional, (6) responsive professional, (7) partnership and (8) empowerment. The previously designed taxonomy of patient values is enriched and slightly adapted by the results of this study. Some new elements were distinguished and illustrated with meaningful statements of participants (Figure 1).

### 3.1 Theme 1: Values of oneself

This theme includes values that reflect the broad ideas of participants about health and healthcare and comprises two elements: (1) uniqueness and (2) autonomy.

#### 3.1.1 Uniqueness

The majority of the participants indicate that personal recognition and the wish to be seen as **a unique individual** is important.

P02 Sometimes specialists make you feel as if they are just checking things off a list … It would be better not to see them as they have already made up their minds about you.

Participants expect **a deep understanding** and acceptance of their personal environment and life choices. Physiotherapists should be able to empathically understand their patients and fully accept their choices.

P13 The therapist asked me questions such as “You have grandchildren, do you play actively with them?”… I replied that I love to play and horse around with them… I was then asked whether this subsequently made me feel unwell again, which it did, but the way I see it is, if I have had a fun day and it made me feel good then I'll suffer the consequences and see how it goes.

As a result, problems like impairments and functional limitations should not be seen as isolated phenomena but as part of a process of suffering in which the patient as a whole entity is involved. Participants recognize that social and psychological factors play an important role in health problems and are willing to share this personal information, provided that it influences care and treatment. They should expose **a holistic vision** on health.

P05 I think it is important that the physiotherapist is aware of how I am in my family and working life and whether or not these factors play a role or create added stress or tension to my situation.

#### 3.1.2 Autonomy

This emerging element was discussed in the interviews as the patient's **right to decide.** The participants want to be well‐informed by the professional in order to make a good decision by themselves or to understand why a certain decision by the professional is the correct one.

P03 That is very important for me… I am not used to passing the initiative or the problem to someone else and then just sitting back and waiting to see what they come up with. I like to be the one who decides…

Respecting the patient's input in treatment and care by physiotherapists is important to all participants. An essential issue mentioned was **independence**.

P01 If you can do things for yourself then you feel less dependent. Feeling dependent is awful, it's always there when you need care but it is good if you can minimalize the feeling.

### 3.2 Theme 2: Values of the professional

This theme reflects the views of participants about professional behaviour and management of practice by physiotherapists which involves also moral‐ethical considerations that go beyond the personal lifeworld of the individual. Four elements could be identified across the manifest data: (1) technically skilled professional, (2) conscientious professional, (3) compassionate professional and (4) responsive professional.

#### 3.2.1 Technically skilled professional

All participants value a physiotherapist who is **competent**, experienced and has **good communicative skills**, such as being open, direct and honest.

P03 Having an experienced person is important. Not someone who just talks the talk, but someone who can really help and knows what they are talking about.

Participants value a thorough analysis and a clear explanation and information on the health problem which requires a **goal‐ and process‐orientated** physiotherapist. Each treatment should be designed for working in a safe, effective and directed way. Prognosis or expectations of treatment should be tailored and adjusted when necessary.

P02 The bursitis was really very difficult and painful in my shoulder. There seemed to be very little progress so a colleague was brought in for a second opinion. The physiotherapist then consulted my GP as to the best remedy to ease the pain.

Both during treatment and in the business operations, the professional should be **positive**.

P17 When someone really shines at what they are doing and they really seem to enjoy their work, then you can see that it's not just about earning money…

P11 This physiotherapist looked at me… and said "It will be better". And then I am pleased.

#### 3.2.2 Conscientious professional

Conscientious behaviour refers to the critical attitude of the practitioner and his moral consciousness in which the patients' interests must prevail. At first, participants value a professional who **acts morally in clinical decision‐making**. The physiotherapist must establish an ongoing commitment to the patient and remain honest, even though the problem or situation is complex. He must not lapse into a routine action and respect his own professional boundaries and honour existing commitments.

P13 In the period when they didn't actually know what was wrong with me I had treatment regardless, I wasn't assessed at all, just continual treatment. Almost ritually. He stopped evaluating if I was doing OK. Then it would be better if he'd just say: I don't know how to proceed. I can't do anything for you anymore.

Secondly, conscientious behaviour refers to **act morally on a commercial and financial level**. Participants think that finance should not override patient interests, and treatment should not simply revolve around health insurance.

P09 All I had to do was call…they always had time for me… The therapist only needed five or ten minutes. The first time I went to another physiotherapist he worked with me for thirty minutes to mobilize me and I thought to myself “What was the other one doing then?” I think it was a question of money…

Lastly, the physiotherapist has to be **in control** and responsible for decision‐making in treatment within his discipline. Participants are of the opinion that loss of control on the part of the physiotherapist due to regulations of their health insurers leads to too much generalization and protocoled treatment at the expense of the quality of individual care.

P06 No, this morning, the physiotherapist filled in an evaluation form to measure any progress saying that there hadn't been any progress at all. I just thought: “What would you do that for?” Because it was a compulsory form which didn't fit my criteria. In my opinion we had achieved a lot.

#### 3.2.3 Compassionate professional

A substantial part of the interviews could be assigned to the element of compassion, which can be understood as a deep sympathy for the patient. The participants value a **concerned** professional who is able and willing to empathize with a person and his or her unique history and questions.

P05 I also felt that there was always time for my questions. There was a lot of attention to detail. Because of this I felt as if I was actually being listened to and heard. This instilled confidence in me to be more open and we were able to get to the core of the problem.

Consultations can result in a deeper relationship by an increased number of consultations or increased consultation length. This allows for a more comfortable situation and a mutual exchange of thoughts by **personal contact** with the physiotherapist.

P05 No, you naturally have a connection with some people. You chat about how your week was etcetera… I had appointments a few times a week, then you don't just talk about your complaints.

To be **taken seriously,** turned out to be a very important patient value. The physiotherapist should not generalize and trivialize the health problem of the patient.

P01 The specialist doesn't have to be too familiar or too amicable. But nothing is worse than a professional who makes you feel as if you are overreacting. This can and does happen.

#### 3.2.4 Responsive professional

Data analyses revealed the importance of a committed and responsible execution of treatment and care by a physiotherapist who **adapt to the patient**s' needs and circumstances. For example; providing information is important, but the professional must adjust the depth of information to the extent of the patient's needs.

P07 Understandably, they don't need to tell me exactly which muscle is which, or be too specific medically. That's not necessary, I would rather it was kept simple.

The physiotherapist should also consider **continuity of care**.

P02 Yes, I don't think that patients should be passed from one professional to another. There should be a valid explanation should this happen. However, this can also be a positive thing as a different perspective can be good.

Participants explicitly mentioned the value of being **aware of vulnerability and dependency**. In contrast to the element independence in Theme 1, this element is focused on the fact that in certain situations patients are dependent on the knowledge and skills of the professional. While the patient value ‘autonomy’ in decision‐making, they also value an awareness on the part of the physiotherapist for the patients' vulnerability when independent decision‐making is not possible.

P01 Patients are often in a vulnerable position because there is something that they are worried but often don't know what it is and they are dependent on the professional.

On the same note, the physiotherapist must **respect the patient's boundaries** concerning his or her personal pain threshold and intimacy. All women interviewed referred to the awareness of incongruity in the patient–provider relationship according to touching, undressing and personal space. Half of the women often felt uncomfortable in their underwear, especially in the presence of men.

P08 Because, to me, what a physiotherapist does can be quite intimate at times. Someone is literally working in your personal space… I remember during the first few treatments I talked a lot and asked a lot of questions to distract from my nakedness. Maybe that is why I began exercise‐based treatments. By keeping my clothes on I felt more comfortable and safe.

Some participants highlighted the possible importance of **cultural sensitivity** by taking into account language barriers and religious differences.

P01 I think this could be quite difficult in clinics where there are a lot of immigrants as they may have difficulties communicating and then more time is necessary to be able to communicate with them to help them properly.

Participants mentioned that the outer appearance of facilities with regard to hygiene and soundness contributes to a sense of **feeling safe**. The practice should be clean, hygienic and in order.

P12 Most important is the quality of care, but the treatment should be carried out in a clean and hygienic environment, not in some scruffy clinic. That gives the right impression and makes you feel more comfortable and safe.

### 3.3 Theme 3: Values of interaction

This theme reflects the process of interaction between the patient and the professional itself, where partnership and empowerment are the core elements. Notably, ‘values of oneself’ and ‘values of the professional’ are intertwined as such as in values for cooperation. The elements in this theme can be distinguished from the other themes by reciprocity and an expectation for a bidirectional effort and commitment.

#### 3.3.1 Partnership

Participants value that interaction with the professionals is based on **equality** and involves mutual respect in an open and understanding ongoing dialogue. They should be able to talk easily and deliberately.

P13 Yes, you would assume that the person treating you is an expert, you should be able to comment if you feel the treatment isn't going according to plan. You should not feel that you are unable to make any comments because you are so dependent on them.

Participants value **cooperation and creating space** for their contribution. Both sides should acknowledge the existing interdependence and must take responsibility.

P03 I like to contribute information by telling my specialist about my activities … whether I have been able to exercise, what went well, what didn't etcetera …

P06 I can't expect the specialist to solve everything alone.

Participants would like to have a certain influence on their treatment, but they expect the professional to **take the lead** in this collaboration due to the professional's superior knowledge.

P06 The discussion is equal, however, I am seeing a professional for their expertise otherwise I wouldn't be seeing them.

P08 Sometimes it is difficult as a patient to understand the problem, medically the professional should be able to do this. You can't do this as a patient.

#### 3.3.2 Empowerment

The majority of the participants' value **empowerment** by the physiotherapist. It enables them to keep control of their own situations and support or educate them in how to deal with the problem. Empowerment also includes professionals who help patients towards self‐management and prevention.

P01 I think it is important that the patient is aware that they have some control over the situation. It is important that the professional encourages you to cooperate in the healing process. Even though they are helping you, you are the most important factor in the process of healing and improvement.

Lastly, the physiotherapist should, among other things, provide tips, tools and exercises to **support** **self‐control**.

P18 That someone explains and demonstrates how and why you have to do the exercises… so that you can go back home motivated to do them.

## 4 DISCUSSION

This study aimed to further substantiate our knowledge about the content of patient values in physiotherapy. The findings of this study show that patient values can be categorized into three themes: (1) values of oneself, (2) values of the professional and (3) values of interaction. These themes can be subcategorized into eight elements: (1) uniqueness, (2) autonomy, (3) technically skilled professional, (4) conscientious professional, (5) compassionate professional, (6) responsive professional, (7) partnership and (8) empowerment. These results are in line with our previous systematic review about the content of patient values in healthcare practices (Bastemeijer et al., 2017). The interviews enriched and expanded on current insights of patient values which has resulted in an adaptation of the preliminary taxonomy. The element ‘professionalism’ is refined by the partitioning into ‘conscientious professional’ and ‘technically skilled professional’ and by renaming the elements compassion and responsiveness. By integrating the results of this study into the taxonomy, the latter was enriched and adapted to the context of physiotherapy practice (Figure 1).

Although patient values are considered important in high value care and are explicitly a part of concepts as EBP, VBP and PCC, they are largely unclear and unknown how to be ‘integrated’ in clinical decision‐making (Charles et al., 2011; Kelly et al., 2015; Rosenbaum, 2013). This vagueness is an important topic to consider as this conceptual flaw may have negative consequences for the quality of physiotherapy practice. Research shows that patients with chronic musculoskeletal pain are often dissatisfied about the quality of the care they receive (Harding et al., 2005; Toye & Barker, 2012). Important aspects of dissatisfaction are the feeling of not being heard, understood or even not being taken seriously (Potter et al., 2003; Trede, 2012). Arguably, our results were not surprising given recent focus on patient‐centred care and mutual decision‐making, but regardless, the findings solidify our assumptions of what patients' value during care. Harnessing the findings in physiotherapy practice might have important consequences for the quality of physiotherapy practice (Bernhardsson et al., 2017; Mudge, Stretton, & Kayes, 2014; Wijma et al., 2017).

The explication of the content of patient values and the categorization of separate values into a taxonomy is important as it may form a background against which physiotherapists (clinicians, researchers, educators) can discuss the implementation of patient values into clinical practice, research and education (van der Weijden et al., 2010). The taxonomy can form a counterweight against an overreliance on scientific evidence as the cornerstone of clinical practice. Although evidence and values are nicely balanced in the definition of ‘EBP’, patient values are easily forgotten, pushed away or seen as less important in daily practice where standardization, objectivity and accountability are all guiding principles for high‐quality care (Epstein, Fiscella, Lesser, & Stange, 2010; Greenhalgh, Howick, & Maskrey, 2014).

This study has some strengths and weaknesses. To our knowledge, this is the first study that aims to describe the content of patient values in physiotherapy practice and to organize these values in a taxonomy. An earlier taxonomy is enriched by integrating the results of this study, enabling further research on this topic. We collected patient values via interviews using an open format, giving participants room to describe the aspects they explicitly value in physiotherapy practice. The pre‐defined taxonomy served as a guide for these interviews but was not used as a compelling tool. Themes of this taxonomy were openly discussed and adjustments could be made. Thematic analyses were carried out by two experienced researchers in the field of physiotherapy and were further discussed with a team to protect the conclusion drawn from implicit bias. Theoretical saturation was used as criterium to end further data collection. Weaknesses of this study involve the choice of including participants with spinal or shoulder pain, which are common health problems in physiotherapy practice, but maybe give an incomplete representation of results.

Further research on the importance of patient values should go in different directions. A first direction is how sensitivity for patient values can be learned during physiotherapy education. Becoming a conscientious, compassionate and responsive professional may require specific educational strategies and training and needs to be addressed accordingly. Second, further research is needed whether the implementation of strategies that harness patient values in daily practices lead to higher perceived quality of care by clients. Third, more theoretical reflections are needed to clarify the relation between scientific knowledge and patient values. The integration of the different kinds of ‘knowledge’ (scientific evidence vs. moral values) cannot easily be integrated and may therefore lead to clinical dilemma's when evidence and the patients' values point in different directions (Epstein et al., 2010; Greenhalgh et al., 2014; Jeffrey & Foster, 2012).

## 5 CONCLUSIONS AND PRACTICE IMPLICATIONS

The findings of this study help physiotherapists to understand what patients with musculoskeletal pain value in physiotherapy practice. Three themes were defined and categorized in a (pre‐existing) taxonomy in order to gather knowledge about the nature of patient values in physiotherapy practice: (1) values of oneself; (2) values of the professional and (3) values of interaction. Two to four elements per theme were identified; uniqueness, autonomy, technically skilled professional, conscientious professional, compassionate professional, responsive professional, partnership and empowerment. This knowledge characterizes individual clinical encounters and may help physiotherapists to be patient‐centred by the integration of moral values in scientific evidence. The results of this study may contribute to further research into this important aspect of the quality of physiotherapy practice and inspire clinicians and educators to actively implement patient values in clinical practice and the education of physiotherapists.

## CONFLICT OF INTEREST

The authors declare no conflict of interest.

## AUTHOR CONTRIBUTIONS

Carla M. Bastemeijer contributed to this study by collecting data through interviews, data analysis and writing all drafts of the study. Johannes P. van Ewijk and Jan A. Hazelzet contributed to this study by approval of data analysis, correcting of all drafts of the study and approved the submitted manuscript. Lennard Voogt contributed to this study by data analysis, correcting of all drafts of the study and approved the submitted manuscript.

## ETHICAL APPROVAL

Ethical approval was given by the Institutional Review Board Erasmus MC Rotterdam in the Netherlands, case number MEC‐2015‐260.
